# A Large Cohort Study of SARS-CoV-2 Detection in Saliva: A Non-Invasive Alternative Diagnostic Test for Patients with Bleeding Disorders

**DOI:** 10.3390/v13122361

**Published:** 2021-11-25

**Authors:** Josiane Iole França Lopes, Carlos Alexandre da Costa Silva, Rodrigo Guimarães Cunha, Alexandra Martins Soares, Maria Esther Duarte Lopes, Orlando Carlos da Conceição Neto, Arthur Daniel Rocha Alves, Wagner Luis da Costa Nunes Pimentel Coelho, Luiz Amorim Filho, Luciane Almeida Amado Leon

**Affiliations:** 1 Instituto de Hematologia Arthur de Siqueira Cavalcanti/Hemorio, Rua Frei Caneca, 8, Centro, Rio de Janeiro CEP 20211-030, Brazil; josiane.iole@hemorio.rj.gov.br (J.I.F.L.); carlosalexandre113@gmail.com (C.A.d.C.S.); rodrigogcunha@gmail.com (R.G.C.); maralexandrasoares@gmail.com (A.M.S.); ester.lopes@gmail.com (M.E.D.L.); orlandocdcnetto@yahoo.com.br (O.C.d.C.N.); luizamorimfilho@gmail.com (L.A.F.); 2 Laboratório de Desenvolvimento Tecnológico em Virologia, Oswaldo Cruz Institute/Fiocruz, Avenida Brasil, 4365, Manguinhos, Rio de Janeiro CEP 21040-900, Brazil; arthuralves@aluno.fiocruz.br (A.D.R.A.); wagnercoelho@aluno.fiocruz.br (W.L.d.C.N.P.C.)

**Keywords:** saliva, SARS-CoV-2, diagnosis, COVID-19, hematological disease, bleeding disorders

## Abstract

Diagnosis of SARS-CoV-2 infections is mostly based on the nasopharyngeal swabs (NPS). However, this collection is invasive and uncomfortable, especially for children and patients with coagulopathies, whose NPS collection often causes bleeding. Thus, the aim of this study was to evaluate the usefulness and accuracy of saliva for the diagnosis of COVID-19 in patients presenting bleeding disorders. Samples of NPS, oropharyngeal swabs (OPS), and saliva were collected simultaneously from 1159 hospitalized patients with hematological diseases and from 524 healthcare workers, both symptomatic and asymptomatic for SARS-CoV-2. All samples were evaluated for SARS-CoV-2 by qRT-PCR. SARS-CoV-2 was detected in NPS, OPS and saliva from 16.9%, 14.4% and 15.6% individuals, respectively. Tests in saliva showed sensitivity, specificity, and overall agreement of 73.3%, 96.9% and 92.7% (=0.74), respectively. Salivary tests had good accuracy (AUC = 0.7) for discriminating negative and positive qRT-PCR for SARS-CoV-2. Higher sensitivity was observed in symptomatic than in non-symptomatic patients, as well as in healthy subjects than in patients with hematological disease, in both OPS and saliva. The mean viral load in NPS was significantly higher than in OPS and in saliva samples (*p* < 0.001). Saliva is a good diagnostic tool to detect SARS-CoV-2, especially among patients symptomatic for COVID-19, and is a valuable specimen for mass screening of hospitalized patients with hematological diseases, especially for those that with bleeding disorders.

## 1. Introduction

COVID-19, caused by severe acute respiratory syndrome coronavirus 2 (SARS-CoV-2), was first described in Wuhan, China, in December 2019. The number of confirmed patients with COVID-19 rapidly grew up in China; thereafter, it spread throughout the world, and the World Health Organization (WHO) declared COVID-19 as a pandemic on the 11th of March 2020.

Common signs and symptoms of COVID-19 include fever, sore throat, cough, malaise, headache, nausea, vomiting, diarrhea, and breathlessness [1]. The clinical manifestations of COVID-19 are widely heterogeneous, ranging from asymptomatic infection to severe pneumonia with respiratory failure and multi-organ dysfunction or death [2,3].

The main route of SARS-CoV-2 transmission is person-to-person contact through respiratory droplets, although there is the possibility of spread by touching the eyes, nose, or mouth after touching contaminated surfaces [3,4,5]. As a support strategy for virus transmission, massive testing of the population and social isolation have been recommended by the WHO [6]. The increase in massive testing capacity imposes the development of new strategies for COVID-19 diagnosis, with non-invasive and self-collection samples of patients.

Although several commercial serological tests are available for the diagnosis of COVID-19, polymerase chain reaction (RT-qPCR) on upper respiratory specimens remains the gold standard for the diagnosis of acute SARS-CoV-2 infections [7]. The collection of specimens by nasopharyngeal swab, however, is not an ideal sample collection procedure for massive screening, as this implies that the patient has to go to an appropriate collection site, such as a clinic or hospital, thus causing the crowding of healthcare institutions. The collection is technically challenging, because it requires close contact between health professionals and patients, and promotes mucosal irritation, leading to coughing, and sneezing, with consequent elimination of viral particles, representing increased occupational risk. Furthermore, it is an invasive and uncomfortable collection process, which makes sample collection even more difficult, especially among children and patients with bleeding disorders. In patients with coagulopathies, such as hemophilia and thrombocytopenia, nasopharyngeal swab collection often causes bleeding, which represents a limitation of great importance mainly for hematological and cancer centers, which need to routinely screen and monitor SARS-CoV-2 infection in their hospitalized patients with hematological disorders.

Saliva has been pointed out by several studies as an alternative specimen to carry out the SARS-CoV-2 detection by RT-qPCR [8,9,10,11,12], since it shows advantages that could overcome many limitations of the respiratory specimen’s collection procedure, such as the non-invasive collection and possibility of self-collection samples. A recent review about salivary use for diagnosis of COVID-19 reported that most salivary diagnostic platforms have been consolidated primarily from letters and reviews [13], indicating a low number of cross-sectional studies. Additionally, the limited number of comparative studies, and lower sample sizes in the studies, compromise the quality and confidence of salivary tests for COVID-19. Another gap concerning the use of saliva for COVID-19 tests is the absence of studies comparing the performance of saliva tests among different population groups, such as asymptomatic vs. symptomatic, coupled with scarce analytical validation studies based on international guidelines [14]. Such analysis could make a substantial difference in the quality of studies of diagnostic accuracy and to provide the best possible evidence about the performance of saliva for SARS-CoV-2 detection.

In this study, samples of saliva from hospitalized patients with hematological diseases and from healthcare workers, both symptomatic and asymptomatic for SARS-CoV-2, were evaluated for SARS-CoV-2 detection through real-time RT-qPCR assays, compared to nasopharyngeal (NPS) and oropharyngeal (OPS) swab tests. All measures of diagnostic accuracy of the saliva tests were evaluated to guarantee a more robust analysis of the methodology.

The aim of this study was to evaluate the usefulness and accuracy of saliva specimens for diagnosis of COVID-19 in patients with bleeding disorders, where non-invasive collection is preferred. Furthermore, we aimed to analyze the performance of saliva tests in asymptomatic and symptomatic subjects, as well as to compare the results with their clinical and laboratory data.

## 2. Materials and Methods

### 2.1. Study Population

From January/2020 to April/2021, all patients admitted consecutively to Hemorio’s wards, and healthcare workers with suspected COVID-19 or with close contact with people who tested positive for COVID-19 were included in this study. Hemorio is specialized in the treatment of high-complexity primary hematological diseases, including oncologic- and non-oncologic hematological diseases, such as coagulopathies- hemophilia, von Willebrand diseases, hereditary thrombocytopathies (about 2600 patients with coagulopathies in follow up at this hospital in 2021), as well as a large number of onco-hematological patients (acute leukemia, lymphoma, etc.) presenting severe or even extreme thrombocytopenia. As a safety protocol to avoid COVID-19 transmission, all patients admitted to this hospital and healthcare workers are routinely tested for COVID-19. Participants’ sociodemographic details and presence or absence of symptoms at the time of sampling were collected. This study was approved by HEMORIO Ethics Commission/IRB (CEP-HEMORIO; No. 4.611.802; CAAE: 40200920.0.0000.5267).

### 2.2. Sample Collection and Processing

Matched samples of nasopharyngeal swabs (NPS), oropharyngeal swab (OPS) and saliva were collected simultaneously from each subject participating of this study, as follows: two NPS samples (one swab for each nostril) containing 3 mL of viral transport medium (VTM) (Laborclin, Paraná, Brazil), one OPS sample containing 3 mL of VTM (Laborclin, Paraná, Brazil), and saliva (spit) were collected using 50 mL sterile sputum containers containing 300 µL of 0.9% NaCl solution.

The collection of NPS and OPS samples was performed by nurses or physicians, while saliva samples were self-collected by each patient, under nurse supervision. NPS collection was performed by rubbing a swab on the posterior part of the nostril, with a rotation movement until the nasopharynx was reached, and the sample was obtained by rotating the swab gently for 5 s.

For the collection of the OPS, the swab was inserted in the oral cavity, then rubbed on the posterior part of the pharynx and regions of the tonsils, avoiding contact with the tongue, teeth, and gums. Then, the NPS and OPS were placed into a 5 mL tube, containing 3 mL viral transport medium (VTM). The samples were immediately transported to the laboratory and the tubes containing the swabs were vortexed vigorously, then VTM was transferred to 1.5 mL tubes and stored at −80 °C until RNA extraction. To self-collect the saliva, patients were requested to avoid eating, drinking, and smoking for at least 30 min prior to spit into the sterile tube containing saline solution (0.9% NaCl). Specimens were considered acceptable if at least 1 mL of saliva has been obtained. The saliva samples were immediately transported to the laboratory and the tubes were vortexed vigorously. Before storage at −80 °C, an aliquot of 300 µL of saliva was transferred to sterile tubes and mixed with 300 µL of VTM, to evaluate whether the SARS-CoV-2 RNA is stable in saliva, comparing the results between preserved and unpreserved saliva.

### 2.3. Detection of SARS-CoV-2

Viral RNA was extracted from 300 µL of NPS, OPS and saliva (with VTM and without VTM), using the automatic MDX Biorobot ^®^ (Qiagen, Hilden, Germany). The detection of SARS-CoV-2 was performed using the Molecular kit SARS-CoV-2 (E) (Bio-Manguinhos/Fiocruz, Rio de Janeiro, Brazil) to detect the RdRp and E genes, based on Charité/Berlim protocol [15], according to manufacturer’s instructions.

One-Step Real-time PCRs were carried out on Taqman ABI 7500 Real Time PCR System^®^ (ThermoFisher, Waltham, MA, USA) using the following cycling conditions: 15 min at 45.0 °C, 02:00 min at 95 °C, followed by 45 cycles of 15 s at 95 °C and 30 s at 58 °C. Fluorescence was collected after each cycle at the annealing/extension step. All samples were run in duplicate. Samples with Ct-values ≤ 40 were considered positive.

### 2.4. Statistical Analysis

The statistical software package GraphPad Prism 5.0 for Windows (GraphPad Software 5.0 version) was used for data analysis. Descriptive analyses of the variables were expressed as mean (±standard deviation = SD), median, or number (%). One-way ANOVA was used to compare NPS, OPS and saliva CT median values. Fisher’s exact test was used to calculate sensitivity, specificity, positive and negative predictive values, and Likelihood Ratio of the diagnosis tests. Agreement between NPS, OPS and saliva was performed using κ statistics. The correlation of Ct values between NPS, OPS and saliva was assessed using Pearson correlation coefficient. AUC (Area under the ROC Curve) was used to analyze the accuracy of saliva tests. In this study, AUC 0.9 to 1 was defined as excellent accuracy, 0.8 to 0.9 as very good, 0.7 to 0.8 as good, 0.6 to 0.7 as sufficient, 0.5 to 0.6 as bad, and <0.5 as poor. A *p*-value < 0.05 was considered statistically significant.

## 3. Results

### 3.1. Studied Population

From January/2020 to April/2021, a total of 1683 individuals were included in this study. The average age of the subjects was 35.59 ± 21.82 years old, ranging from 1 to 96 years, and most of them were female (59%) (Table 1). Among them, 1159 (68.9%) were patients with hematological diseases, and 524 (31.1%) were healthcare workers. None of the healthcare workers had hematological diseases. Of patients with hematological diseases, 97 had coagulopathies. Table 2 shows the coagulopathies, according to the risk of bleeding during the swab collection: high risk (platelets < 20.000 mm^3^), moderate (platelets 20.000–50.000 mm^3^), and low risk (platelets > 50.000 mm^3^). SARS-CoV-2 was detected in 71.4% (10/14) of those with a severe risk of bleeding. Other hematological diseases were present in 943 patients, such as bone marrow aplasia (n = 12), sickle cell disease (n = 378), Gaucher disease (n = 1), Hodgkin’s disease (n = 12), non-Hodgkin’s disease (n = 66), lymphoproliferative disease (n = 4), hereditary spherocytosis (n = 3), Acute myeloid leukemia (n = 91), acute lymphoid leukemia (n = 216), chronic lymphoid leukemia (n = 104), multiple myeloma (n = 34), thalassemia (n = 1), agranulocytosis (n = 1), aplastic anemia (n = 6), iron deficiency anemia (n = 1), and autoimmune hemolytic anemia (n = 13).

Clinical symptoms of COVID-19, such as fever, sore throat, headache, cough, and breathlessness, were reported by 645 (38.3%) participants. Among them, 481 (74.6%) were healthcare workers, while 164 (25.4) were patients with hematological disease (Table 1). Matched samples of NPS, OPS and saliva were obtained from 1440 participants.

### 3.2. Detection Rate of SARS-CoV-2 in NPS, OPS and Saliva

**Figure 1:**
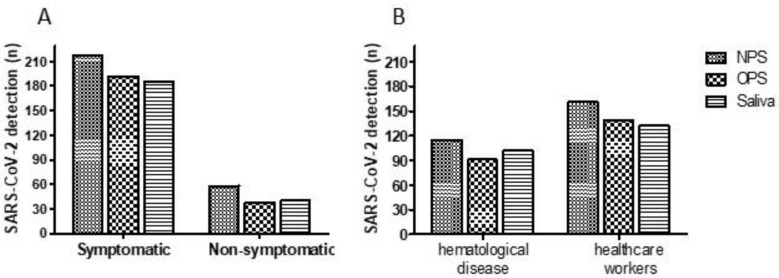
SARS-CoV-2 detection according to the studied population. (**A**) SARS-CoV-2 detection according to the presence of symptoms. (**B**) SARS-CoV-2 detection between patients with hematological disease and healthcare workers. NPS: nasopharyngeal swab, OPS: oropharyngeal swab.

Of the 1683 subjects screened for SARS-CoV-2, 300 were positive in at least one of the specimen types (NPS, OPS, and saliva), which results in an overall positivity rate of 17.8%. Out of 1629 participants screened for SARS-CoV-2 in NPS samples, 276 (16.9%) were positive. Most of them were healthcare workers (n = 161, 58.3%), and showed symptoms (n = 218, 78.9%) (Figure 1). The frequency of SARS-CoV-2 detection in OPS and saliva was 14.4% (230/1596) and 15.6% (234/1498), respectively (Table 3).

Among paired samples of NPS, OPS and saliva obtained from 1440 subjects, the overall positivity of SARS-CoV-2 was 257 (17.0%), 220 (15.3%) and 225 (15.6%), respectively. Of 1440 subjects with paired samples tested, 94.3% were positive in two or more tests, while 53 (3.7%) were positive only in one type of specimen. In 36 (68%) of these 53 subjects, only the NPS was positive, in 2 (3.8%) only de OPS was positive, and only in 20 (37.73%) was the saliva positive.

Symptomatic subjects showed significantly higher viral detection rate in NPS, OPS and saliva (78.9%, 83.5% and 79.5%, respectively), compared with those asymptomatic for SARS-CoV-2 (21%, 16.5%, 17.1%, respectively) (*p* < 0.001) (Table 3). In the paired samples from symptomatic individuals, SARS-CoV-2 was detected in 207 NPS samples and in 185 saliva samples, of which 160 individuals had both samples test positive, while 381 individuals were negative in both tests, which resulted in 541 of 612 matches (overall agreement of 88.4%). Out of 821 paired samples from asymptomatic individuals, viral RNA was detected both in NPS and saliva in 27 individuals, and 759 were negative in both tests, which resulted in 778 of 821 matches (overall agreement of 95.7%).

In all types of specimens, a significantly higher SARS-CoV-2 rate of detection was shown among healthcare workers (58.3% in NPS, 60.4% in OPS and 56.4% in saliva) as compared to patients with hematological diseases (41.7% in NPS, 39.6% in OPS and 43.6% in saliva) (*p* < 0.001). A significant difference was not observed between the detection rate of SARS-CoV-2 in NPS vs. saliva, or between NPS and OPS, according to the clinical characteristics of the studied population (Table 3).

### 3.3. Validation Parameters of Saliva and OPS for Detection of SARS-CoV-2

Validation parameters of the saliva and OPS tests were evaluated among paired samples (n = 1440), using NPS as a reference gold standard. Comparing SARS-CoV-2 detection in OPS and NPS, a total of 208 subjects tested positive in both specimens, whereas 10 samples was positive only in OPS, which represents a sensitivity and specificity of 81.5% and 99.2%, respectively. There was an overall agreement of 96% (1369/1426) between NPS and OPS (κ = 0.85; 95% CI 0.76–0.93, *p* < 0.0001), indicating an almost perfect agreement (Table 4).

Diagnostic tests in saliva samples showed a sensitivity and specificity of 73.3% and 96.9%, respectively, since 188 paired samples were positive in both specimens (NPS and saliva), and 38 were positive only in saliva samples. Among OPS tests, a sensitivity of 81.5% and specificity of 99.2% were observed. The overall agreement between NPS and saliva samples was 92.7% (1354/1461) (κ = 0.74; 95% CI 0.63–0.77, *p* < 0.0001). Analysis of saliva samples containing VTM (saliva-VTM) revealed that 170 patients tested positive both by NPS and saliva-VTM, showing a sensitivity of 66.1% (95% CI: 0.60–0.72) and specificity of 98% (95% CI: 0.97–0.98), yielding a PPV of 88% (95% CI: 0.82–0.92) and NPV of 92.9% (95% CI: 0.91–0.94) (Table 4).

**Figure 2:**
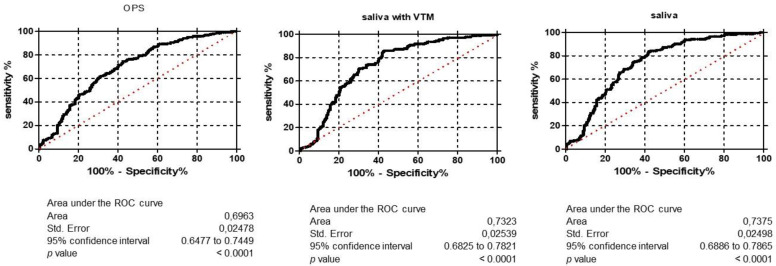
Area under the ROC curve (AUC) of OPS, saliva and saliva with VTM. NPS: nasopharyngeal swab, OPS: oropharyngeal swab, saliva—VTM: saliva with viral transport medium. Ct: cycle threshold.

AUC was analyzed to estimate the discriminative power of the saliva and OPS tests for SARS-CoV-2 detection. Figure 2 shows the area under the ROC curve of OPS (AUC = 0.69), saliva (AUC = 0.70), and saliva-VTM (0.71), indicating that these tests had good accuracy in discriminating health condition (negative qRT-PCR for SARS-CoV-2) from disease condition (positive qRT-PCR for SARS-CoV-2).

**Figure 3:**
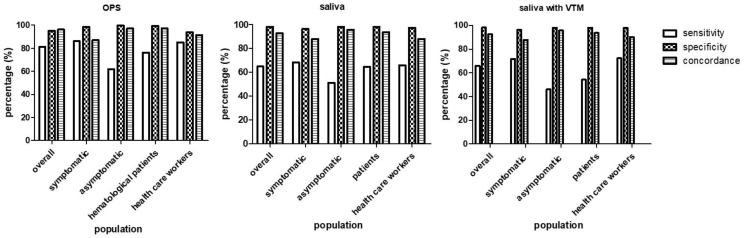
Accuracy of OPS and saliva tests according to the studied population, compared with NPS (the gold standard method). OPS: oropharyngeal swab, saliva-VTM: saliva with viral transport medium.

The accuracy of saliva and OPS tests varied according to the studied population. Comparing the performance of the saliva and OPS tests between symptomatic and non-symptomatic individuals, both tests showed higher sensitivities among symptomatic (78% and 86%, respectively) than non-symptomatic (55% and 61.8%) subjects. Higher sensitivities, in both saliva and OPS tests, were also seen among healthcare workers (72.3% and 85%, respectively) compared to patients with hematological disease (64.4% and 76.3%, respectively) (Figure 3).

### 3.4. Quantitative Detection of SARS-CoV-2 in Saliva as Compared with NPS and OPS

**Figure 4:**
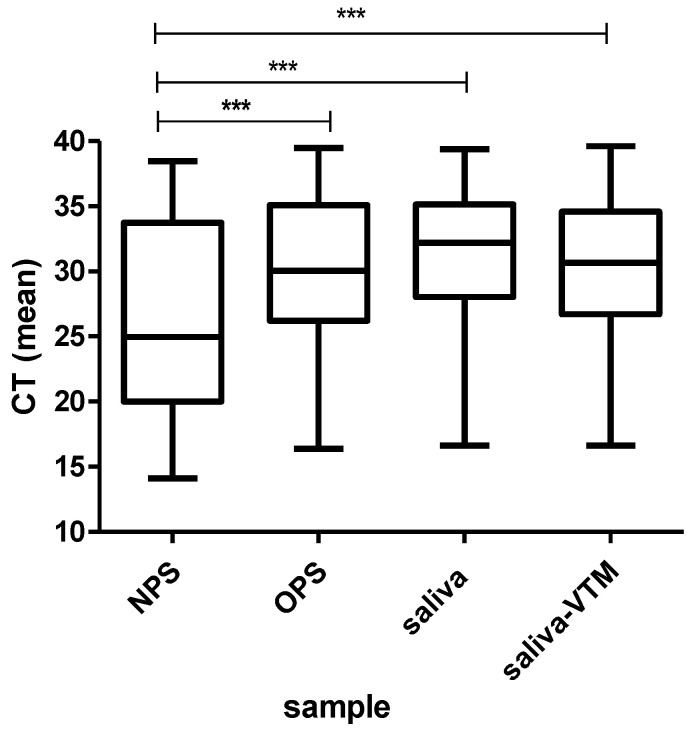
Mean of SARS-CoV-2 viral load in NPS, OPS and saliva samples. NPS: nasopharyngeal swab, OPS: oropharyngeal swab, saliva-VTM: saliva with viral transport medium. Ct: cycle threshold. *** *p* < 0.001.

**Figure 5:**
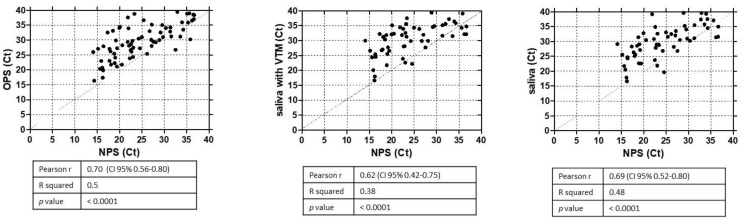
Comparative Ct-values scatter plots showing the correlation between NPS and different samples collection methods. Each dot represents one patient. Pearson’s correlation coefficient indicates the strength of linear dependence between the different types of samples.

The median of cycle threshold (Ct) in NPS (24.9) was significantly lower than in OPS (30.1) and in saliva samples (32.1) (*p* < 0.001) (Figure 4). Scatter plot and regression analysis were performed to show the correlation between NPS and saliva Ct values (Figure 5). The Pearson’s correlation coefficient of 0.69 indicates that viral load detected in saliva was correlated with that observed in NPS. The mean Ct in saliva samples was significantly lower among symptomatic patients (29.5) than in asymptomatic patients (33.6) (*p* = 0.012). In contrast, any significant difference was observed between mean Ct values of the saliva from healthcare workers (29.4) and patients with hematological diseases (31.15) (*p* = 0.22).

## 4. Discussion

This study reported for the first time the accuracy of SARS-CoV-2 detection using saliva as compared to NPS and OPS in a large group (n = 1683) of patients with hematological diseases and healthcare workers. In our study, the SARS-CoV-2 RNA overall detection rate was 16.9% with NPS and in 15.6% with saliva samples, which indicates that saliva is a promising biological matrix for detection of SARS-CoV-2.

Saliva tests showed good specificity (97%) and sensitivity (73%). Previous studies reported that the sensitivity of RT-qPCR–analyzed saliva specimens varied from 42.6% to 97% for SARS-CoV-2, as compared with the nasopharyngeal swabs [16,17]. The difference in sensitivities probably reflects differences in the sample collection procedure, timing of sampling in each study and sample sizes. Although high sensitivities of saliva tests have been reported, low sample sizes from the published studies seem to be a limitation, compromising the evaluation of the actual diagnosis value of saliva [10,17,18,19,20,21]. Yokoto et al. [22] also showed a higher sensitivity (92%) in a larger size study (n = 1924 individuals); however, this study analyzed the accuracy and sensitivity of saliva using a Bayesian latent class mode instead of comparing saliva results with nasopharyngeal swabs as a gold standard.

Besides a substantial agreement between saliva specimens and NPS (92.7%, κ = 0.73), saliva tests showed a positive likelihood ratio (LR = 23.4), which means that is more likely that saliva positive test result will occur in subjects with COVID-19 than in subject without this disease. In this study, the overall accuracy of saliva tests was also expressed as area under the ROC curve (AUC), since AUC provides a useful parameter for comparing tests performance [23,24]. Saliva tests showed AUC of 0.73, which is a good indicator of the quality of the test, demonstrating that it is able of discriminating between diseased and non-diseased. Together, the validation parameters demonstrate that saliva can be a good alternative diagnostic tool for the detection of SARS-CoV-2.

Some issues regarding collection and processing of saliva are critical and could lead to discrepancies between the study’s results [13]. Thus, we compared the performance between saliva with VTM as preserving, and unpreserved saliva. In this study, this protocol reduced the sensitivity to 66%, as compared to 73% in saliva without VTM, indicating that this dilution of saliva interferes with the result, probably due to the changes in the SARS-CoV-2 concentration.

Viral load in saliva was significantly lower than in OPS and NPS. In fact, several studies have reported reduced viral load in saliva [25], which reflects the lower rate of SARS-CoV-2 detection in this specimen. In accordance with previous studies [26,27], saliva had a higher detection rate and lower Ct-value (high viral load) among symptomatic individuals than in asymptomatic, which is probably reflected in the lower sensitivity of the test among the asymptomatic patients. A similar finding has been reported for NPS, where the sensitivity of this test is higher in symptomatic than in asymptomatic COVID-19 subjects [28]. However, the specificity of 98% suggests the reliable detection limits of the current assay for detecting the absence of SARS-CoV-2 viral loads in saliva samples even in asymptomatic individuals. Although a significantly higher SARS-CoV-2 rate of detection was observed among healthcare workers compared with patients with hematological disease, this finding was probably due the fact that most of the healthcare workers were symptomatic for COVID-19, since we did not observe any significant difference in mean Ct-values among these studied groups.

We also highlight that 53 patients tested positive for SARS-CoV-2 by RT-qPCR in only one type of sample. Among them, 38% (20/53) only tested positive in saliva, which corresponds to a 6.67% saliva-only detection rate. Similar findings of substantial salivary detection of SARS-CoV-2 in patients with a negative nasopharyngeal swab has been reported [11,29,30], suggesting that specimens from multiple sites may improve the sensitivity and reduce false-negative test results. In addition, the presence of SARS-CoV-2 RNA only in saliva analyzed by RT-qPCR must not be classified as false positive, but it could be related to the limitations in nasopharyngeal swab procedure, or due to the time of NPS sampling, leading to false negative results. Savela et al. [25] showed that SARS-CoV-2 RNA first appears in saliva and then in nasal-swab samples, with a high-sensitivity detection of SARS-CoV-2 in saliva 1.5 to 4.5 days before the viral load in the paired nasal-swab samples exceeded the limit of detection. Therefore, it must be considered that neither saliva nor NPS have a 100% sensitivity.

Our data confirm that a reliable detection of SARS-CoV-2 especially among symptomatic COVID-19 patients can be obtained with saliva, and its diagnostic performance is comparable to the current standards (nasopharyngeal swabs). On the other hand, salivary diagnosis showed a limitation regarding COVID-19 asymptomatic patients, but it is important to emphasize that the sensitivity of diagnostic COVID-19 test among asymptomatic patients remains unclear in nasopharyngeal specimens [13].

Although our results showed that, in general, saliva is less sensitive when compared to NPS and OPS, the collection of these two respiratory specimens are not an easy or an ideal method for widespread screening and may be associated with various degrees of discomfort for the patient [31]. This can be more dangerous for patients having thrombocytopenia or any other coagulation disorder [32], in which the sample collection can cause bleeding. In our study, among patients with coagulopathies (n = 97), 14 (14.4%) had level of platelets below 20,000/mm^3^, which is considered a severe risk of bleeding. Most of them were SARS-CoV-2 positive in NPS (10/14), OPS (10/14), and in saliva (9/14). No significant difference was observed in the detection rate of SARS-CoV-2 between NPS, OPS and saliva samples from patients with coagulopathies.

We tried to establish the feasibility of the use of saliva for diagnosis of SARS-CoV-2, mainly for patients with hematological disorders, as saliva can easily be collected by the patient, and it is a non-invasive collection. Accordingly, it seems reasonable to incorporate the saliva-based SARS-CoV-2 assay into a part of multiple lines of diagnostics, which believably may further facilitate the identification of COVID-19 patients, especially for those with coagulopathies.

## Figures and Tables

**Table 1:** Demographic data of the studied population.

	Total (n, %)	Hematological Diseases (n, %)	Healthcare Workers (n, %)
n (%)	1683 (100)	1159 (68.9)	524 (31.1)
Age (mean ± SD)	35.59 ± 21.82	33.57 ± 24.97	39.43 ± 15.08
Gender			
Female	993 (59)	592 (51.1)	394 (77.7)
Male	690 (41)	567 (48.9)	113 (22.4)
Symptomatic ^1^	645 (38.3)	164 (25.4)	481 (74.6)
Non-symptomatic	1035 (61.5)	999 (96.5)	36 (3.5)

^1^ Clinical symptoms of COVID-19 were considered such as fever, sore throat, headache, cough, and breathlessness.

**Table 2:** Patients with coagulopathies and other hematological diseases.

	Total	Platelets (<20,000)	Platelets (20,000–50,000)	Platelets (>50,000)	NPS (n, %)	OPS (n, %)	Saliva (n, %)
Coagulopathies ^1^ (n)	97	14	7	71	10 (10.3)	10 (10.3)	9 (9.3)
*Hemophilia A*	36	0	0	35	25 (69.4)	24 (66.6)	24 (66.6)
*Hemophilia B*	9	0	0	7	4 (44.4)	6 (66.6)	6 (66.6)
*Von Willwbrand disease*	1	0	0	1	1 (100)	1 (100)	1 (100)
*Essential thrombocytopenia*	11	0	1	10	11 (100)	11 (100)	8 (72.7)
*Thrombocytopenia purple*	32	14	6	9	31 (96.8)	30 (93.7)	29 (90.6)
*Factor VII deficiency*	1	0	0	1	1 (100)	1 (100)	1 (100)
*Hemoglobinopathy*	2	0	0	2	2 (100)	2 (100)	2(100)
*Hereditary thrombopathy*	3	0	0	3	3 (100)	3 (100)	3 (100)
*Paroxysmal Hemoglobinuria*	1	0	0	1	1 (100)	1 (100)	1 (100)
*Paroxysmal Myoglobinuria*	1	0	0	1	1 (100)	1 (100)	1 (100)
*Glanzan Disease*	1	0	0	1	1 (100)	1(100)	1 (100)
Other hematological diseases ^2^	943	2	6	935	90 (9.5)	70 (7.4)	65 (6.9)

^1^ Two coagulopathy patients without platelets information; ^2^ 216 patients with hematological diseases without platelets information.

**Table 3:** Comparison of SARS-CoV-2 detection in saliva, NPS and OPS according to clinical characteristics of the population.

	NPS (n = 1629)	Saliva (n = 1498)	*p*-Value (NPS and Saliva)	OPS (n = 1596)	*p*-Value (NPS and OPS)
Positive samples (n, %)	276 (16.9)	234 (15.6)	0.45	230 (14.4)	0.76
Age (mean ± SD)	42.8 ± 16.5	41.5 ± 15.8	0.46	43.4 ± 15.9	0.74
Gender					
Female	191 (69.2)	160 (68.4)	0.61 (0.80–1.12)	161 (70.0)	0.52 (0.73–1.08)
Male	85 (30.1)	74 (31.6)	0.61 (0.80–1.12)	69 (30.0)	0.52 (0.73–1.08)
Symptomatic ^1^	218 (78.9)	186 (79.5)	0.81 (0.58–1.45)	192 (83.5)	0.81(0.80–1.16)
Non-symptomatic	58 (21.0)	40 (17.1)	0.81 (0.58–1.45)	38 (16.5)	0.81(0.80–1.16)
Patients with hematological diseases	115 (41.7)	102 (43.6)	0.93 (0.86–1.18)	91 (39.6)	0.65 (0.88–1.22)
Healthcare workers	161 (58.3)	132 (56.4)	0.93 (0.86–1.18)	139 (60.4)	0.65 (0.88–1.22)

^1^ Clinical symptoms of COVID-19 were considered such as fever, sore throat, headache, cough, and breathlessness.

**Table 4:** Validation parameters of the saliva and OPS tests compared with NPS (gold standard method).

Sample Type ^1^	qPCR Positiven (%)	Agreement (%)	Sensitivity (95% CI)	Specificity (95% CI)	PPV (95% CI)	NPV (95% CI)	Kappa	AUC (95% CI)	LR
OPS	220 (15.3)	96.12	81.5 (0.80–0.89)	99.2% (0.98–0.99)	94% (0.90–0.97)	97% (0.96–0.97)	0.85	0.69 (0.68–0.78)	85.68
Saliva	225 (15.6)	92.70	73.3 (0.67–0.78)	96.9% (0.95–0.97)	83% (0.78–0.88)	94% (0.92–0.95)	0.74	0.73 (0.66–0.75)	23.38
Saliva with VTM	193 (13.4)	92.28	66.1 (0.60–0.72)	98% (0.97–0.98)	88% (0.82–0.92)	92.9% (0.91–0.94)	0.71	0.73 (0.68–0.78)	33.6

^1^ Number of paired samples was 1440. OPS: oropharyngeal swab; Saliva with VTM: saliva with viral transport medium; qPCR: quantitative PCR; PPV: positive predictive value; NPV: negative predictive value; AUC: area under the curve; LR: likelihood rate; 95% CI: 95% confidence interval.
